# Increasing young adults’ condom use intentions and behaviour through changing chlamydia risk and coping appraisals: study protocol for a cluster randomised controlled trial of efficacy

**DOI:** 10.1186/1471-2458-13-528

**Published:** 2013-05-30

**Authors:** Katie V Newby, David P French, Katherine E Brown, Donna M Lecky

**Affiliations:** 1 Applied Research Centre in Heath and Lifestyle Interventions (ARC-HLI), Coventry University, Priory Street, Coventry CV1 5FB, UK; 2 School of Psychological Sciences, University of Manchester, Manchester M13 9PL, UK; 3 Public Health England Agency Primary Care Unit, Microbiology Department, Gloucester Royal Hospital, Great Western Road, Gloucester GL1 3NN, UK

**Keywords:** Randomized controlled trial, Chlamydia, Risk appraisal, Coping appraisal, Sexual behaviour, Condom use, Motivational hypothesis

## Abstract

**Background:**

Chlamydia is the most commonly diagnosed sexually transmitted infection (STI) in England and has serious public health consequences. Young people carry a disproportionate burden of infection. A number of social cognition models identify risk appraisal as a primary motivator of behaviour suggesting that changing risk appraisals for STIs may be an effective strategy in motivating protective behaviour. Meta-analytic evidence indicates that the relationship between risk appraisal and health behaviour is small, but studies examining this relationship have been criticised for their many conceptual and methodological weaknesses. The effect of risk appraisal on health behaviour may therefore be of larger size. The proposed study aims to examine the efficacy of an intervention to increase condom use intentions and behaviour amongst young people through changing chlamydia risk and coping appraisals. Coping appraisal is targeted to avoid the intervention being counterproductive amongst recipients who do not feel able to perform the behaviour required to reduce the threat. An experimental design with follow-up, a conditional measure of risk appraisal, and analysis which controls for past behaviour, enable the relationship between risk appraisal and protective behaviour to be accurately assessed.

**Methods/Design:**

The proposed study is a two-arm cluster randomised controlled trial using a waiting-list control design to test the efficacy of the intervention compared to a control group. Participants will be school pupils aged 13–16 years old recruited from approximately ten secondary schools. Schools will be randomised into each arm. Participants will receive their usual teaching on STIs but those in the intervention condition will additionally receive a single-session sex education lesson on chlamydia. Measures will be taken at baseline, post-intervention and at follow-up three months later. The primary outcome measure is intention to use condoms with casual sexual partners.

**Discussion:**

As far as the authors are aware, this is the first controlled trial testing the efficacy of an intervention to increase condom use intentions and behaviour through changing chlamydia risk appraisals. It is one of few experimental studies to accurately test the relationship between risk appraisal and precautionary sexual behaviour using a conditional measure of risk appraisal and controlling for past behaviour.

## Background

Chlamydia is the most commonly diagnosed sexually transmitted infection in England [1] and also across Europe [2]. It is frequently asymptomatic [3], and without treatment can lead to serious health consequences for women such as pelvic inflammatory disease, infertility and ectopic pregnancy. There is also growing evidence that it can cause infertility in men [4,5].

There is evidence that young people, who are disproportionately affected by chlamydia [6], may be underestimating the risk of infection. Studies have for example identified that important knowledge gaps exist [7-9] and that unhelpful beliefs may be influencing chlamydia risk appraisals in ways that reduce motivation to adopt protective behaviour [8].

A number of social cognition models include risk appraisal as a primary motivator of behaviour. These include the health belief model [10], the precaution adoption process [11] and protection motivation theory [12]. These models suggest that changing risk appraisals could be an effective strategy in motivating protective behaviour such as condom use.

The motivational hypothesis [13] asserts that preventative behaviour is the result of the desire to reduce one’s risk. It makes logical sense that to be motivated to take precautionary behaviour one has to perceive the consequences of inaction as serious and likely to occur. Systematic reviews examining the predictive relationship between risk appraisal and health behaviour have however largely found this relationship to be small [14,15], or absent in the case of sexual behaviour specifically [13]. This suggests that an intervention aiming to increase chlamydia risk appraisals will have either no effect or a weak effect on condom use intentions and behaviour.

The existing body of evidence however suffers serious problems which may have served to underestimate the relationship between risk appraisal and future behaviour. Following a review of nearly 60 studies examining this relationship, Weinstein and colleagues [16] concluded that a high proportion had serious conceptual and methodological flaws. These included the failure to control for past behaviour and the use of correlational data to examine the relationship between risk appraisal and behaviour. The use of correlational data to examine the relationship appears to be particularly common in studies of sexual behaviour [13]. Although cross-sectional designs always limit conclusions that can be drawn regarding cause-effect relationships, they are especially problematic when examining the motivational hypothesis. This is because risk appraisals are both a determinant and a product of risk behaviour. According to the motivational hypothesis, it is the belief that taking precautionary behaviour will be effective in reducing feelings of risk that motivates an individual to act. It follows that once preventative action is taken, feelings of risk are reduced. This means that when taking concurrent measures of risk appraisal and behaviour, a negative relationship should be expected. For this reason, Weinstein and colleagues [16] advise using longitudinal data, where risk appraisal is measured at time one, behaviour is measured at time two, and analysis controls for behaviour at time one.

A further methodological problem is that studies examining the motivational hypothesis have largely used ‘unconditional’ measures of risk. Unconditional measures are those which ask individuals to rate the probability of an adverse event occurring without indicating, for example, whether this is if they do or do not use a condom, whether this is with a long-term partner or a casual sexual partner. Using conditional measures of risk is preferable, as these questions elicit risk appraisals based on such moderating factors. Conditional measures of risk are not only more conceptually accurate but enable the relationship between risk and behaviour to be examined in a consistent and interpretable manner [17]. Weinstein and colleagues [16] advise that when testing the motivational hypothesis, that conditional measures of risk are taken in which individuals are asked to either rate their perceived vulnerability to the health threat if they continue with their existing levels of behaviour or if they take no precautionary action.

The present study will assess the efficacy of an intervention to change risk appraisals which overcomes the problems of much previous work in this area. The intervention is a single Sex and Relationships (SRE) lesson produced for the Health Protection Agency (HPA) and made available to teachers across ten European Union countries on their e-Bug website. The lesson was developed using Intervention Mapping [18,19] which provides a framework for developing theory- and evidence-based interventions. A detailed report on the development and content of the intervention can be accessed using this link (http://www.healthinterventions.co.uk/projects.aspx?section=10&item=78).

In addition to strategies to increase chlamydia risk appraisals, the chlamydia lesson incorporates strategies to raise condom use response efficacy (the perceived effectiveness of condoms in reducing the threat of chlamydia) and self-efficacy (beliefs about one’s own ability to use condoms). Protection Motivation Theory (PMT) [12] was developed based on the observation that increasing risk appraisals amongst individuals who do not believe that they are able to perform behaviour can be ineffective. PMT predicts that as individuals’ feelings of threat increase, that protective behaviour will also increase if they feel able to cope with that threat. If on the other hand they feel they can do nothing or little to change their behaviour, then increased perceptions of risk can be counter-productive leading to avoidance (e.g. avoid thinking about STIs), information derogation (e.g. ‘health related messages are over-hyped’), or threat minimisation (e.g. denying you’re at risk of STIs). Evidence concerning whether threat appraisal and coping appraisal (the combined effect of response and self-efficacy) interact or operate in parallel is inconclusive [15]. Until the nature of this relationship has been substantiated, interventions should seek to raise both threat and coping appraisals.

The proposed study uses a two-arm cluster randomised controlled trial (RCT) with a waiting-list control to test the efficacy of an intervention in increasing young adults’ condom use intention and behaviour through changing their chlamydia risk and coping appraisals. This will provide useful information about the ability of persuasive communication to bring about the desired programme effects. The waiting list control design has been chosen to allow all participants to benefit from the lesson. Cluster randomisation has been chosen to prevent within-school contamination.

The proposed study will use a conditional measure of risk. Given that a large proportion of the sample are not likely to be sexually experienced, participants appraisals of the risk of getting chlamydia if condoms aren’t used will be measured, rather than choosing the alternative ‘if continue with current behaviour’ option. In addition, this will be framed within the context of casual sexual partners for whom STI status is unknown given that this presents the most risky scenario. A further conditional stipulation will be vaginal sex, rather than oral or anal sex. The proposed study will also use an experimental design with follow-up for two months. Examining the motivational hypothesis in the context of an experimental study is ideal since delivery of the intervention should act to ‘destabilise’ existing chlamydia risk appraisals and reduce the predictive relationship between past and future behaviour for those who are already sexually experienced [16].

Whilst an experimental design affords the best circumstances for observing the motivational hypothesis, increasing chlamydia risk appraisals may not be sufficient to motivate condom use. Chlamydia is a single consequence of unprotected sex and evidence suggests that other outcomes of unprotected sex, such as the risk of pregnancy or making sexual experiences more enjoyable, may exert a more powerful effect on condom use intentions [20]. Other authors [13] have also drawn attention to the dyadic context of condom use decision making and the possibility that risk appraisals are not powerful enough to overcome other determinants of behaviour such as embarrassment discussing condom use or uncooperative partners. The proposed study will include a measure of condom use intention as well as behaviour. This is necessary because it is anticipated that a large proportion of the sample will be sexually inexperienced requiring the use of intention as the outcome measure in examining the motivational hypothesis. In addition, it will also enable examination of whether an increase in chlamydia risk appraisals is sufficient to increase condom use intentions and/or behaviour. This will enable conclusions to be drawn about whether a failure to change behaviour is the result of a failure to increase condom use intentions or to overcome the effect of stronger factors operating when intention is translated into behaviour.

### Research questions

#### Primary research question

•Is the lesson effective in increasing young people’s *intentions to use* condoms during vaginal sex with casual sexual partners?

#### Secondary research questions

•Is the lesson effective in increasing young people’s *condom use* during vaginal sex with casual sexual partners?

•If the lesson is effective, are changes in young people’s condom use intentions or behaviour due to changes in their chlamydia risk appraisals (perceived likelihood and severity) and/or coping appraisals (condom use response efficacy and self-efficacy)?

### Ethical review

This study has received ethical approval from Coventry University Ethics Committee.

## Methods/design

**Figure 1:**
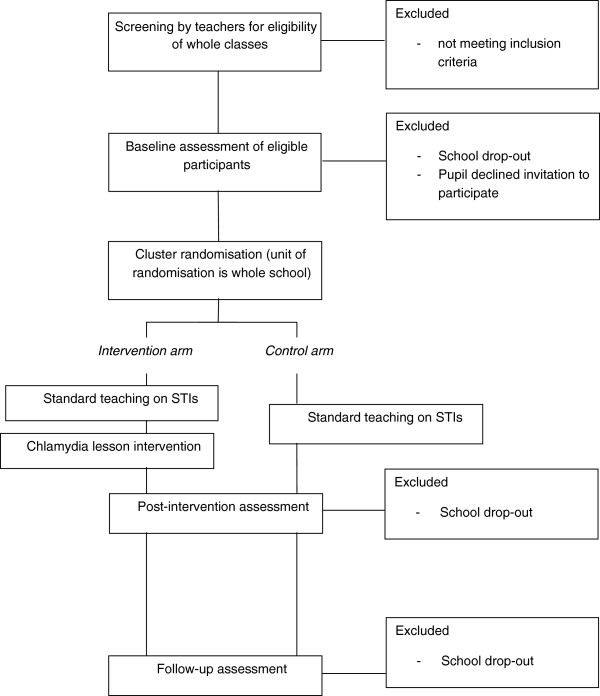
Study design.

A two-arm cluster randomised controlled trial (RCT) using a waiting-list control will be used to test the efficacy of an intervention to increase young adults’ intentions to use condoms and actual use of condoms with casual sexual partners compared to a control group (see Figure 1). Secondary school pupils will be invited to participate. The intervention is a single lesson on chlamydia which aims to increase young adults’ chlamydia risk and coping appraisals. We hypothesise that there will be an increase in condom use intentions following delivery of the chlamydia lesson. At the two month follow-up, we hypothesise that there will be a higher rate of condom use during vaginal sex with casual sexual partners amongst sexually experienced participants in the intervention group compared with the control group. At two months, we will also measure condom use intentions to see whether any increases have been maintained over time. We hypothesise any effects of the intervention on condom use intentions or behaviour will be mediated by risk appraisals and/or coping appraisals.

### Participant recruitment and baseline assessment

The study will take place in approximately ten schools across England. All schools will be comprehensive secondary schools and have a standard Sex and Relationships (SRE) policy and SRE curriculum in place.

Researchers will make contact with SRE co-ordinators at the schools who will describe the study and invite participation. SRE co-ordinators who wish to participate will be required to obtain permission from the school head teacher. They will be asked to identify classes receiving SRE in their school where pupils are aged 13–16 years old. SRE teachers must confirm that the chlamydia lesson has not previously been delivered to these pupils and that they have not received any teaching on STIs. Full details on the requirements of participating schools will be provided. Schools will be offered a £100 Amazon voucher in recognition of the time and resource commitment required. They will also be offered an individual school report based on anonymised and aggregated pupil data from this study which may be helpful in informing their SRE policy and curriculum.

Participating SRE teachers will schedule one lesson (45 minutes) per class to provide study information, obtain consent and collect baseline measures. Pupils will be told that the study aims to evaluate teaching on STIs in their school, but not informed that there are two intervention arms. Pupils will also be told about the number and nature of assessments that will be made, and how their data will be protected and treated. They will be provided with a written participant information sheet and asked to sign a consent form.

Pupils will not be obliged to take part. Quizzes will be administered at the same time as questionnaires at all data collection time points to occupy pupils who do not wish to participate. Pupils will not be required to provide their name (participant responses at each time point will be linked through the demographic information supplied). Pupils will be seated in a manner that prevents classmates from overseeing their responses i.e. in exam conditions. All materials (questionnaires and quizzes) will be placed in a sealed envelope by the individual participants and posted into sealed boxes on completion. Teaching staff will not have access to these. Although parental consent will not be required, as parents of participating pupils will have already consented for their children to participate in SRE and related evaluation activities, a letter outlining the study and their child’s involvement will be sent to all parents through each school as a matter of courtesy. Parents will be given the opportunity to ‘opt-out’ their child at this point.

Baseline measures will be: Demographic information (age, date of birth, gender, and ethnicity), perceived likelihood, perceived severity, response efficacy, self-efficacy, past behaviour, and intention to use condoms during vaginal sex with casual sexual partners for whom STI status is unknown. Provisional measures to be taken at baseline, post-intervention and follow-up are provided in Additional file 1.

After the collection of baseline data, schools will be randomised to receive either the school’s usual teaching on STIs and the chlamydia lesson (intervention arm) or the school’s usual teaching on STIs alone (control arm). This enables the study to evaluate the effectiveness of the chlamydia lesson over and above usual practice. As described above, pupils will be blinded to intervention condition. It is not possible to blind teachers to intervention condition but they will be made aware of the importance of delivering teaching that is consistent with their usual practice. Researchers liaising with schools will offer the same guidance and level of support regardless of intervention condition.

As with all trials of this type, there is the potential for drop-out of whole schools during the study. We have no way of estimating the level of this. Drop-out due to unforeseen circumstances will be planned for and a reserve list of schools will be created to expedite the process of additional recruitment if required.

### Randomisation and allocation concealment

To avoid contamination, we will use cluster randomisation using schools as the randomisation unit. Schools will be allocated to either arm of the study using block randomisation with a block size of four. This will be performed by an independent researcher. Group allocation will only be revealed to participants following the collection of baseline data.

### Sample size calculation

The calculation is based on the primary research question. Power analysis using GPower 3.1.2 has indicated that 484 participants will provide 95% power to detect a small to medium effect of 0.3 [21] where α = 0.05. The effect size of 0.3 is based on a recent meta-analysis examining the effectiveness of interventions in changing risk appraisals, intentions and behaviour [22]. Due to the tendency for individuals within a cluster to be more similar to each other than those in other clusters, it is necessary to make adjustments to avoid an overestimation of power [23]. In order to obtain this adjusted sample size, the sample size determined by usual procedures is inflated by multiplication with a factor. This factor is known as the “design effect” and is calculated as:

$$1+\left(n–1\right)\mathrm{ICC}$$

where *n* is the average cluster size, and ICC is the intraclass correlation coefficient. The ICC for intention to use condoms between schools is not known but an estimate of 0.01 is reasonably conservative [23]. With ten schools and an average cluster size of 60 (×2 classes of 30 pupils), the design effect is:

$$1+\left(60–1\right)0.01=1.59$$

Accordingly the adjusted sample size is 484 × 1.59 = 770. We will over-recruit to allow for drop-out between the start of the study and the three month follow-up.

### Intervention

The intervention comprises a 45 minute lesson which is freely available in the form of a lesson plan and associated resources from the Health Protection Agency’s e-Bug website. E-bug is a school resource available across 10 EU countries covering microbes, their prevention, spread and treatment, including the spread of Sexually Transmitted infections. The lesson consists of four semi-interactive exercises, three of which are delivered using animated resources. These are accompanied by a lesson plan. The lesson aims to raise awareness of how chlamydia infection spreads internally causing damage in the short and long term. It challenges false reassurance provided by the belief that infection is easily treated by emphasising that treatment is unlikely to be sought in the absence of symptoms. It encourages pupils to personalise the true prevalence of infection, and highlights the fallibility of using overt characteristics to judge the risk posed by sexual partners. The lesson conveys the effectiveness of using condoms in preventing infection, and provides guidance on how to resist pressure to have unwanted sex, negotiate condom use, and correctly use condoms.

Behaviour Change Techniques (BCTs) used to deliver the lesson are:

•Information about health consequences

•Salience of consequences

•Anticipated regret

•Prompts/cues

•Instruction on how to perform a behaviour

•Demonstration of the behaviour

Whilst appropriate BCTs to target the selected determinants were identified at the time of intervention development using the most detailed and refined taxonomy available at that time [24], the BCTs above are those identified through a process of retrospective selection based on recent guidance (v0.1, 12.10.11) made available to the first author [personal communication; provided by University College London BCT Taxonomy team]. This guidance is based on the Behaviour Change Wheel method for intervention design [25]. The guidance refers to the most refined generic taxonomy available to date [26]. Work to develop this taxonomy is described in Michie and colleagues’ study protocol [27].

Further detail on the development and content of the lesson has been published elsewhere (link). Interested readers are directed to this report for further information.

Lessons in the control and experimental groups will be delivered by their usual timetabled SRE teacher. Those delivering the chlamydia lesson will be given minimal instruction with regards to delivery as all necessary information is contained within the lesson plan. Fidelity to the lesson plan will be assessed using a self-report checklist completed post-delivery (see Additional file 2).

On study completion, all schools allocated to the control group will be directed to the e-Bug website to enable them to download and deliver the chlamydia lesson if they wish providing the findings do not indicate that the lesson has any detrimental effects i.e. unfavourable changes in any of the primary or secondary outcome measures.

### Measures post delivery and at follow-up

Immediately following the lessons, a further set of measures will be taken (see Additional file 1). These will be a repeat of those taken at baseline. For those in the intervention arm, a few additional items will be included to allow for process evaluation. These items will measure participants’ enjoyment of the lesson, collect their opinions on the suitability and relevance of the content for their age, and ascertain the extent to which they felt they could relate to the characters and examples given. It will also determine whether participants felt the materials were engaging, and whether the delivery was sufficiently interactive. Items will be a mixture of fixed response and open-ended formats and will allow any necessary improvements to be made to the lesson following evaluation.

At follow-up, three months post delivery, measures of intention and use of condoms (during vaginal sex with casual sexual partners for whom STI status is unknown) will be taken (see Additional file 1).

A number of the lesson exercises are available as ‘games’ on the HPAs e-Bug website. For this reason, it will be necessary to ensure that those in the intervention group have not viewed these prior to delivery of the lesson, and that those in the control group have not viewed these both before or during the study period. Although we believe this to be unlikely, it will be necessary to determine this to ensure that contamination has not occurred and to control for any dose effect. This will be ascertained using a simple question within the follow-up measure. The reason for not choosing to screen for this at the time of baseline data collection is that doing this may inadvertently draw attention to existence of the ‘games’ and result in participants viewing them out of curiosity resulting in contamination. Data from individuals who viewed the resources prior to delivery of the lesson (intervention group) or before/during study period (control group) will be removed from the analysis.

All questionnaire items will be piloted with approximately five young people prior to use to assess ease of comprehension and the suitability of language. Changes will be made accordingly.

### Outcomes

#### Primary outcome

Intention to use condoms during vaginal sex with casual sexual partners over the next two months.

#### Secondary outcomes

Use of condoms during vaginal sex with casual sexual partners over two months post delivery.

Perceived likelihood of chlamydia.

Perceived severity of chlamydia.

Response efficacy for condom use.

Self-efficacy for using condoms during vaginal sex with casual sexual partners over the next two months.

### Statistical analysis

Prior to analysis, school codes will be removed from the data set by an independent researcher and intervention condition coded as A or B. This will blind analysts to intervention condition. Intention to treat analysis will be used so that data is used regardless of whether pupils are present or absent during the STI and chlamydia lessons in either of the intervention arms, and means are imputed for pupils with missing post-delivery and/or follow-up data. Whole classes or schools who drop-out during the study and don’t provide at least post-delivery data will be removed from the analysis.

#### To examine the effect of the intervention

Analysis of Covariance (ANCOVA) will be performed to examine differences in condom use intentions (at time two and at follow-up), and behaviour. This will control for demographic variables (gender, ethnicity, age), behaviour at time one, and measures of risk appraisal, and coping appraisal.

If the lesson is effective in increasing condom use intentions, mediation analysis [28] will be conducted to identify whether changes in risk appraisals and/or coping appraisals are responsible for this change. This will help to identify the mechanism of change and indicate where future research and/or resources should be focussed.

## Discussion

This study protocol presents the design of a cluster randomised controlled trial testing the efficacy of a SRE lesson intervention to increase young adults’ condom use intention and behaviour through enhancing chlamydia risk and coping appraisals. As far as the authors are aware, this is the first controlled trial testing the efficacy of an intervention to increase condom use intentions and behaviour through modifying chlamydia risk appraisals, and one of few studies to accurately test the motivational hypothesis in the context of precautionary sexual behaviour.

A limitation of this study is that sexual behaviour will be measured by self-report. Clearly the use of self-reported condom use is unavoidable given the sensitive and private context of the behaviour. Test-retest reliability analyses and validation of self-reports against reports from sexual partners suggest however that, despite the potential for biased reporting, self-report measures of condom use do have satisfactory reliability and validity [29-31]. A further limitation of this study is the focus on chlamydia risk appraisal in isolation. Evidence suggests that other sources of perceived risk, such as the risk of pregnancy are likely to be driving intentions to engage in protective sexual behaviour [20]. This study therefore does not examine or account for, the relative contribution of other sources of risk which may be equally or more powerful predictors of intention. Finally it should be noted that there is a follow-up period of only two months. Whilst a short-term follow-up is typical of trials evaluating the efficacy of sexual health behaviour change interventions [32] it means that long-term efficacy will not be established by this study.

If the intervention is effective in increasing risk appraisal, response efficacy or self-efficacy, this study will provide important information about which behaviour change techniques can be used to bring about favourable changes in these behavioural determinants for precautionary sexual behaviour. If the intervention is effective in changing condom use intentions or behaviour, then it can be recommended for widespread distribution within schools. It also has the potential for use within alternative contexts such as by the National Chlamydia Screening Programme (NCSP) to encourage uptake of screening invitations. If the findings of the proposed study support the motivational hypothesis, then this will support the continued inclusion of risk appraisal within theories of health behaviour change.

## Competing interests

The author(s) declare that they have no competing interests.

## Authors’ contributions

KN conceived and designed the study. KN, DF, and KB drafted the manuscript. All authors read and approved the final manuscript.

## Authors’ informations

KN is a Research Fellow and HCPC registered Health Psychologist.

DF is a professor of Health Psychology and BPS chartered psychologist.

KB is a Reader in eHealth and Wellbeing interventions, leads a research group studying adolescent sexual health, and is a BPS chartered psychologist, and a HCPC registered Health Psychologist.

DL is a microbiologist and e-Bug project manager for Public Health England Agency’s Primary Care Unit.

## Pre-publication history

The pre-publication history for this paper can be accessed here:

http://www.biomedcentral.com/1471-2458/13/528/prepub

## Supplementary Material

Additional file 1 Provisional questionnaire items. Click here for file

Additional file 2 Teacher checklist and feedback form. Click here for file
