# Supplementary material for: Increasing young adults’ condom use intentions and behaviour through changing chlamydia risk and coping appraisals: study protocol for a cluster randomised controlled trial of efficacy
Source: BMC Public Health. 2013 May 30;13:528. doi: 10.1186/1471-2458-13-528 (PMC3680015; doi:10.1186/1471-2458-13-528)
Supplement: Additional file 1 — Provisional questionnaire items. [file 1471-2458-13-528-S1.docx]

**Evaluation of your Sex and Relationships Education about Sexually Transmitted Infections (STIs)**

**Questionnaire**

Instructions

Please read these notes carefully before you begin.

Within this questionnaire we are interested in what influences your decisions. Please read each question carefully and answer it as truthfully as you can - sometimes people will choose answers that they think others would want them to or would find most acceptable but we need to know how **you really think and feel**. Remember that your answers will be kept strictly private (confidential). We have seated you so that it is difficult for others to see what you are writing. There are no correct or incorrect responses; we are simply interested in your personal point of view.

Throughout this questionnaire, the word ‘sex’ is used to refer to **vaginal sex** (also known as sexual intercourse; penis in vagina). If you think or know that you are homosexual (also known as gay, lesbian etc) then please do not complete the questionnaire. We are sorry that you are not able to take part at this time.

**Casual sexual partner**:

For the purposes of this study we want you to answer questions about how you would think and act if you had sex with a casual sexual partner. You may plan on never having sex with a casual sexual partner, that doesn’t matter, we are just interested in what you think about this. When answering, we want you to picture a casual sexual partner as someone who you have sex with either as a **one-night-stand or on a more regular basis, but are not serious** about. Imagine that you **do not know** whether this person has any sexually transmitted infections.

**Chlamydia** – some of the questions below ask you about chlamydia. This is a sexually transmitted infection (STI) which can be passed through vaginal sex.

**School Name:** *(to be printed on)*

Likelihood measure (taken at all time points)

1. If you were to have sex with a casual partner, how likely do you think you would be to get chlamydia if you **didn’t use** a condom?

Response options: no chance to certain to happen; 7 point scale

1. If you were to have sex with a casual partner, how vulnerable do you think you would be to getting chlamydia if you **didn’t use** a condom?

Response options: not at all to extremely; 7 point scale

Severity measure (taken at all time points)

1. How serious do you think it would be if you were to get chlamydia?

Response options: no harm at all to extremely devastating; 7 point scale

Response efficacy (taken at all time points)

1. If used correctly, condoms are effective in preventing chlamydia

Response options: strongly agree to strongly disagree; 7 point scale

Self-efficacy (taken at all time points)

1. I feel confident that I could buy condoms without feeling embarrassed
2. I feel confident in my ability to discuss condom use with a casual sexual partner
3. I feel confident in my ability to suggest using condoms with a casual sexual partner
4. I feel confident in my ability to persuade a casual sexual partner to use a condom
5. I feel confident in my ability to insist on using condoms before sex
6. I feel confident in my ability to put a condom on myself or my partner
7. I feel confident in my ability to use a condom correctly

Response options: Strongly disagree, disagree, undecided, agree, strongly agree

Intentions – (taken at all time points)

For the next set of questions, we are interested in the opinions of everyone, whether they have had sex or not. If you have not had sex, or don’t think that you would ever have sex with a casual sexual partner, we are still interested in how you think you would feel about using condoms in this situation. Please just imagine that this is a possibility and tell us what you think you would do.

*REMEMBER: you are not indicating here whether you think you are likely or unlikely to have sex with a casual partner but instead whether if you did, how likely you would be to use condoms.*

1. I plan to use condoms every time I have sex with a casual sexual partner

Response options: extremely unlikely to extremely likely; neither likely or unlikely midpoint; 7 point scale

1. I will try to use condoms every time I have sex with a casual sexual partner

Response options: extremely unlikely to extremely likely; neither likely or unlikely midpoint; 7 point scale

1. I intend to use condoms every time I have sex with a casual sexual partner

Response options: extremely unlikely to extremely likely; neither likely or unlikely midpoint; 7 point scale

Sexual experience (taken at baseline only)

1. Have you ever had sex?

Response options:

Yes – p*lease go to question 16*

*No – please go to question X*

1. Have you had sex with a casual sexual partner within the last two months?

Response options:

Yes – please go to question 17

No - *please go to question X*

Past behaviour (taken at baseline)

1. How often have you used condoms when having sex with casual sexual partners over the past two months?

Response options: every time, almost every time, sometimes, almost never, never

Behaviour (taken at follow-up only)

1. Have you had sex since the chlamydia lesson?

Response options:

Yes – please go to question 19

No – please go to question X

1. Have you had sex with a casual sexual partner since the chlamydia lesson?

Response options:

Yes – please go to question 20

No – please go to question X

1. How often have you used condoms when having sex with casual sexual partners since the chlamydia lesson?

Response options: every time, almost every time, sometimes, almost never, never

Demographics (taken at all time points)

1. Are you:

Response options: Male/female

1. What is your date of birth?

Response option: DD/MM/YYYY

1. How old are you?:

Response options: 13/14/15/16/17

1. How would you describe your ethnicity?

Response options: (use census categorisations)

1. What is your mother’s maiden name? (Note: we are only collecting this information to help us link the answers you provide in each of the questionnaires)

Response option: Open text box

**Contamination/dose check (taken follow-up only; intervention schools only)**

*Intervention group:*

During the lesson you received on chlamydia you were shown a number of animations. Please answer the following questions about each:

‘Pants’ animation:

Image here from ‘pants’ animation

Had you seen this before the chlamydia lesson?

Yes/no

Have you viewed any of these again since the lesson?

- No, I have not viewed it since the lesson
- Once
- Two or more times

‘Chlamydia - the movie’ animation:

Image here from ‘chlamydia – the movie’ animation

Had you seen this before the chlamydia lesson?

Yes/no

Have you viewed any of these again since the lesson?

- No, I have not viewed it since the lesson
- Once
- Two or more times

‘Jeopardy’ animation:

Had you seen this before the chlamydia lesson?

Image here from ‘jeopardy’ animation

Yes/no

Have you viewed any of these again since the lesson?

- No, I have not viewed it since the lesson
- Once
- Two or more times

**Contamination check (taken at follow-up only; control schools only)**

Below are images from three different animations which teach about chlamydia available on a website called e-bugs [www.e-bugs.eu](http://www.e-bugs.eu). Please answer the questions about each.

Image here from ‘pants’ animation

‘Pants’ animation:

Have you ever seen this before?

Yes/no

Image here from ‘chlamydia the movie’ animation

‘Chlamydia – the movie’ animation:

Have you ever seen this before?

Yes/no

Image here from ‘jeopardy’ animation

‘Jeopardy’ animation:

Have you ever seen this before?

Yes/no

**Thank you for taking the time to complete this questionnaire**
