# Supplementary material for: Increasing young adults’ condom use intentions and behaviour through changing chlamydia risk and coping appraisals: study protocol for a cluster randomised controlled trial of efficacy
Source: BMC Public Health. 2013 May 30;13:528. doi: 10.1186/1471-2458-13-528 (PMC3680015; doi:10.1186/1471-2458-13-528)
Supplement: Additional file 2 — Teacher checklist and feedback form. [file 1471-2458-13-528-S2.docx]

**Teacher checklist and feedback form**

Your name:

Name of school

Date lesson delivered:

Year group (9, 10 or 11):

Class name/number:

Number in class:

**Checklist**

Please complete the checklist below to indicate which elements of the lesson you delivered according to the lesson plan (please do this by placing ticks in the relevant boxes). Please answer honestly; your responses are simply to provide an indication of consistency amongst teachers delivering the lesson and also to identify any important changes that need to be made. You are given the opportunity to explain reasons for not including or changing specific elements overleaf if you wish to do so.

Introduction

Exercise one ‘pants’

Exercise two ‘If chlamydia could talk’

Exercise three ‘Chlamydia – the movie’

Exercise four ‘Jeopardy’

**Comments**

Please use the space below to comment on any problems/difficulties you had in delivering this lesson (e.g. poor reception of activities, problems with resource materials, time limitations etc.). Any recommendations for changes that you can think of would also be most welcome.

Please continue overleaf if necessary.
